# Lentiviral GLP-1 gene therapy elicits developmental stage–dependent β-cell regeneration in diabetic rats

**DOI:** 10.1007/s00109-026-02690-w

**Published:** 2026-06-12

**Authors:** Ezgi Erbasan, Melike Aliciaslan, Fulya Erendor, Sumeyye Sensoy, Busra Cetin, Mustafa Aydemir, Salih Sanlioglu

**Affiliations:** 1 Department of Gene and Cell Therapy, Faculty of Medicine, Akdeniz University, Antalya, 07058 Turkey; 2 Division of Endocrinology and Metabolism, Department of Internal Medicine, Faculty of Medicine, Akdeniz University, Antalya, 07058 Turkey

**Keywords:** Beta cell differentiation, Gene delivery, Glucagon-like peptide 1, Lentivirus, Type 2 diabetes mellitus

## Abstract

Pancreatic β-cell differentiation and regenerative capacity differ markedly between developmental stages, with the neonatal pancreas exhibiting high plasticity that enables ongoing progenitor- and ductal-derived β-cell formation, whereas the adult pancreas demonstrates limited neogenic potential. Glucagon-like peptide-1 (GLP-1) promotes β-cell survival, proliferation, and differentiation; however, its developmental stage-specific effects on β-cell regeneration are not fully understood. To investigate this, we generated a third-generation HIV-based lentiviral vector encoding native GLP-1 (LentiGLP-1) under the control of cytomegalovirus (CMV) promoter using the Multisite Gateway^®^ recombination cloning system. The vector’s ability to modulate β-cell differentiation and proliferation was subsequently assessed in neonatal and adult diabetic rat models. Type 2 Diabetes (T2DM) was induced in neonatal rats by administering low-dose streptozotocin (STZ), exploiting the intrinsic plasticity of the developing pancreas, whereas in adult rats, a high-fat diet combined with low-dose STZ was used. LentiGLP-1 administration markedly promoted differentiation of ductal and progenitor cells into insulin-producing β-cells in neonatal rats, accompanied by enhanced β-cell proliferation, demonstrating effective engagement of developmental plasticity. In adults, LentiGLP-1 partially restored β-cell populations through activation of residual progenitors and stimulation of replication in existing β-cells, improving glycemic control and insulin sensitivity. Notably, acinar cells did not contribute to β-cell generation in either neonatal or adult models. These results indicate that GLP-1 exerts developmentally regulated effects on β-cell differentiation, facilitating neogenesis in neonates and partially restoring regenerative capacity in adults. Long term GLP-1 expression, thus represents a promising strategy to restore β-cell mass by proliferation and differentiation, providing insight into its therapeutic potential for diabetes.

## Introduction

Pancreatic β-cell differentiation is a highly orchestrated developmental process that begins during embryogenesis and continues postnatally to establish and maintain functional β-cell mass throughout life. In the embryonic pancreas, endocrine cells arise from multipotent progenitor cells (MPCs) located within the foregut endoderm, which sequentially differentiate into endocrine, acinar, and ductal lineages under the control of transcription factors, including pancreatic and duodenal homeobox 1 (*Pdx-1*), neurogenin-3 (*Ngn3*), NK6 homeobox 1 (*Nkx6.1*), and MAF BZIP transcription factor A (*MafA*) [1, 2]. During this period, signaling events within the pancreatic epithelium, such as Notch, Wnt, and FGF pathways, determine lineage specification and endocrine commitment. The neonatal stage represents a transitional period characterized by extensive β-cell proliferation and maturation, during which islet organization and insulin secretory responsiveness are progressively refined [3]. In contrast, the adult pancreas displays markedly limited regenerative capacity. Following β-cell loss or injury, regeneration in adults primarily occurs through the replication of pre-existing β-cells rather than neogenesis from progenitor or ductal cells [4]. However, emerging evidence suggests that under certain physiological or pharmacological stimuli, the adult pancreas retains some plasticity that allows partial reactivation of developmental programs reminiscent of embryonic differentiation [5]. Understanding and harnessing this residual regenerative potential remains a major challenge in diabetes research, as progressive β-cell loss and dysfunction are hallmarks of T2DM [6].

Among molecules implicated in β-cell development and regeneration, Glucagon-like peptide-1 (GLP-1) has gained significant attention due to its dual metabolic and cytoprotective functions. Beyond its well-established role as an incretin hormone, GLP-1 has been shown to influence pancreatic morphogenesis and β-cell lineage determination [7]. Studies have demonstrated that GLP-1 receptor (GLP-1R) activation can stimulate β-cell proliferation, inhibit apoptosis, and induce neogenesis from progenitor cell populations [8, 9]. For instance, exendin-4, a potent GLP-1R agonist, has been reported to upregulate *Pdx-1* and *Ngn3* expression in rodents, promoting β-cell differentiation and increasing islet mass [10]. Furthermore, GLP-1 has been reported to promote endocrine transdifferentiation of pancreatic ductal cells into β-like phenotypes in various pancreatic cell lines [11]. These findings suggest GLP-1 signaling acts as a molecular bridge between metabolic regulation and cellular reprogramming within the pancreas.

Despite these advances, the mechanisms by which GLP-1 modulates β-cell differentiation and regeneration across developmental stages remain incompletely understood. Whether β-cell differentiation triggered by GLP-1 in the neonatal pancreas recapitulates normal developmental processes or engages unique regenerative pathways has not been fully elucidated. Moreover, it remains unclear whether sustained GLP-1 expression can overcome the inherent regenerative limits of the adult pancreas or primarily amplifies existing proliferation. The transient nature of pharmacological GLP-1 analogs and their systemic metabolic effects further complicate the identification of mechanisms underlying β-cell differentiation [12].

To address these gaps, the present study was designed to elucidate the developmental stage–specific effects of GLP-1 on β-cell differentiation and neogenesis. A third-generation HIV-based lentiviral vector encoding native GLP-1 under a CMV promoter (LentiGLP-1) was constructed by using the Multisite Gateway^®^ system to achieve stable, sustained in vivo GLP-1 expression. Two complementary diabetic models were used: a neonatal streptozotocin (STZ) model exploiting the intrinsic plasticity of the developing pancreas, and an adult high-fat diet/low-dose STZ model reflecting the characteristics of T2DM. Through comparative histological and molecular analyses, this study aimed to elucidate how long-term GLP-1 expression influences β-cell differentiation, and progenitor activation in distinct developmental contexts, thereby investigating lentiviral GLP-1 gene delivery as a potential long-term therapeutic strategy for diabetes.

## Materials and methods

### Construction of GLP-1 encoding lentiviral vectors via multisite gateway recombination

A synthetic human *GLP-1* minigene (7–37; 193 bp) was directionally cloned into the pENTR/D-TOPO vector, as described in our previous study [13]. The confirmed GLP-1 entry clone was recombined with a CMV promoter using the Multisite ViraPower™ HiPerform™ Promoterless Gateway™ Vector Kit (Invitrogen, A11146) into the pLenti6.4/R4R2/V5-DEST backbone via Gateway^®^ LR Clonase^®^ II mix. Recombinant constructs were screened by restriction enzyme analysis and DNA sequencing (BigDye™ Terminator v3.1; Applied Biosystems, Foster City, CA, USA).

### Production, purification, and titration of lentiviral vectors

Third-generation lentiviral particles encoding GLP-1 (LentiGLP-1) or LacZ (LentiLacZ) were produced and purified according to the previously established protocol [14]. Briefly, LentiGLP-1 was produced by co-transfecting the transfer vector pLentiCMV-GLP1 with the packaging plasmids pGag-Pol (Addgene, 12251), pRSV-Rev (Addgene, 12253), and pVSV-G (Addgene, 12259). Similarly, the control vector LentiLacZ was generated using the pLacZ plasmid (Addgene, 12108) with the same packaging plasmids.

The functional titer of lentiviral preparations was determined by quantitative PCR (qPCR). qPCR was performed using primers targeting the Woodchuck Hepatitis Virus Posttranscriptional Regulatory Element (WPRE; Forward: 5′-CCGTTGTCAGGCAACGTG-3′; Reverse: 5′-AGCTGACAGGTGGTGGCAAT-3′) to quantify viral genomes, with primers against human albumin (Forward: 5′-GCTGTCATCTCTTGTGGGCTGT-3′; Reverse: 5′-ACTCATGGGAGCTGCTGGTTC-3′) as an internal control. A standard curve was generated from serial dilutions of an albumin plasmid (Addgene). Reactions were carried out using the QuantiTect SYBR Green PCR Kit (Qiagen, 204143) on an ABI 7500 Fast Real-Time PCR System (Applied Biosystems).

### In vitro transduction efficiency of lentiviral vectors

For in vitro transduction efficiency, HepG2 cells (1 × 10⁵/well, 24-well plates) were transduced with LentiGLP-1 or LentiLacZ. Seventy-two hours post-transduction, cells were washed, fixed at room temperature, permeabilized with PBS-T, and blocked with 1% BSA. Cells were then incubated overnight with an anti–GLP-1 primary antibody (Abcam, ab22625) at 4 °C, followed by incubation with Alexa Fluor Plus 488–conjugated goat anti-rabbit IgG (Invitrogen, A32731) at room temperature. Nuclei were counterstained with DAPI, and fluorescence images were obtained using an Olympus IX81 motorized inverted microscope.

### GLP-1 Enzyme-Linked Immunosorbent Assay (ELISA)

GLP-1 secretion following LentiGLP-1 transduction was evaluated in HepG2 cells and in both models. For in vitro analysis, HepG2 cells (5 × 10⁴/well, 24-well plates) were transduced with LentiGLP-1, or LentiLacZ and supernatants were collected 72 h post-transduction. For in vivo analysis, blood from sacrificed rats was treated with a DPP-4 inhibitor and centrifuged at 2500 rpm for 10 min to obtain serum. Active GLP-1 levels in serum and cell culture supernatants were measured using the Glucagon-Like Peptide-1 (Active) ELISA kit (Millipore, EGLP-35 K) according to the manufacturer’s instructions.

### Glucose-Stimulated Insulin Secretion (GSIS) assay

MIN6 cells (1 × 10⁵/well, 24-well plates) were transduced with LentiGLP-1 or LentiLacZ. One day after transduction, the culture medium was replaced with fresh DMEM, and cells were further incubated for 3 days at 37 °C in a 5% CO₂ incubator. For GSIS, cells were washed and incubated in KRBH containing 0 mM, 2.8 mM, or 25 mM glucose. Supernatants were collected, while parallel cultures were incubated overnight at 4 °C in 100% Ethanol/0.4 M HCl to determine total cellular insulin content. Both supernatants and lysates were analyzed using the Ultrasensitive Mouse Insulin ELISA kit (Crystal Chem, Inc., Elk Grove Village, IL, 90080).

### Establishment of type 2 diabetic animal models and gene transfer

Two experimental rat models were established to evaluate the effects of LentiGLP-1 gene delivery: a neonatal STZ-induced diabetic model and an adult obese-diabetic (OD) model. Newborn and adult Sprague Dawley (SD) rats were obtained from Animal Care Laboratory of Akdeniz University Hospitals and maintained in compliance with the guidelines approved by the Institutional Animal Care and Use Committee of the Akdeniz University School of Medicine.

For the neonatal model, SD newborns were divided into two groups. Within 48 h of birth, one group received a single intraperitoneal (i.p.) injection of STZ (100 mg/kg), while the control group received PBS. Animals were sacrificed at defined post-lentiviral injection time points, and blood and abdominal organs were collected. Upon confirmation of successful model induction, additional neonatal rats were allocated into experimental groups for gene transfer experiments. Two groups received STZ (100 mg/kg) at 48 h postnatal, followed 24 h later by i.p. administration of LentiGLP-1 (10¹¹ TU/mL) or LentiLacZ (10¹¹ TU/mL), while controls received PBS. Animals were subsequently sacrificed at the same post-vector injection time points for sample collection.

For the adult OD model, six-week-old male SD rats were divided into two groups. One group was maintained on a standard diet (control), while the other received a high-fat diet (60% kcal from fat) for 2 months, followed by a single i.p. STZ injection (40 mg/kg). Body weight and blood glucose were monitored for 1 month, prior to sacrifice and sample collection. After confirming successful model establishment, additional SD rats (6 weeks old) were divided into four groups: standard diet-fed controls, obese controls receiving PBS, OD rats treated with LentiGLP-1 (10¹¹ TU/mL), and OD rats treated with LentiLacZ (10¹¹ TU/mL). Diabetes was confirmed if blood glucose exceeded 250 mg/dL on both days 3 and 5 post-STZ. Lentiviral vectors were administered 7 days after STZ injection. Body weight and blood glucose were monitored for 1 month, followed by sacrification and sample collection.

### Assessment of blood glucose, glucose tolerance, and insulin sensitivity

In adult OD rats, blood glucose was monitored after STZ administration using an Optima glucometer. Prior to sacrifice, glucose tolerance and insulin sensitivity were assessed using intraperitoneal glucose tolerance tests (IPGTT) and insulin tolerance tests (ITT) respectively. Following fasting, adult OD rats received either glucose (2 g/kg, i.p.) or Humalog insulin (1 U/kg, i.p.), and blood glucose levels were measured at multiple time points thereafter.

### Histological analysis

Abdominal organs (pancreas, kidney, liver, and spleen) were collected from both models and were embedded in paraffin cassettes. Hematoxylin and eosin (H&E) staining was performed on pancreatic and hepatic sections to assess tissue morphology. Immunohistochemical staining was conducted using rabbit polyclonal anti-insulin (Abcam, ab63820) and rabbit polyclonal anti-GLP-1 (Abcam, ab22625). Beta-cell area was quantified by calculating the ratio of insulin-positive area to total pancreatic area using ImageJ software. Imaging was performed with an Olympus IX81 inverted fluorescence microscope.

### Lineage analysis of insulin-positive cells

After standard deparaffinization and hydration, pancreatic sections underwent antigen retrieval in sodium citrate buffer (pH 6.0), followed by permeabilization in ice-cold methanol and 0.25% Triton X-100 in PBS. Non-specific binding was blocked using UV-blocking solution. For dual immunofluorescence, primary antibodies were applied in 1% BSA as follows: polyclonal rabbit anti-insulin (Abcam, ab63820) combined with one of Ki67 (Thermo Scientific, PA5-16446), Ngn3 (Thermo Scientific, PA5-102658), CK20 (Thermo Scientific, PA5-86167), PDX-1 (Thermo Scientific, PA5-78024), or pancreatic alpha-amylase (Thermo Scientific, PA5-78771). Secondary antibodies used were Goat anti-Mouse IgG (H + L) Alexa Fluor™ Plus 488 and Donkey anti-Rabbit IgG (H + L) Alexa Fluor™ Plus 555. Sections were counterstained with DAPI. Imaging was performed using an Olympus IX81 motorized inverted fluorescence.

### Statistical analysis

All statistical analyses were performed using GraphPad Prism 10 (GraphPad Software, La Jolla, CA, USA). Data are presented as mean ± SEM. Statistical significance was defined as *p* < 0.05.

## Results

### Construction and production of the LentiGLP-1

**Fig. 1:**
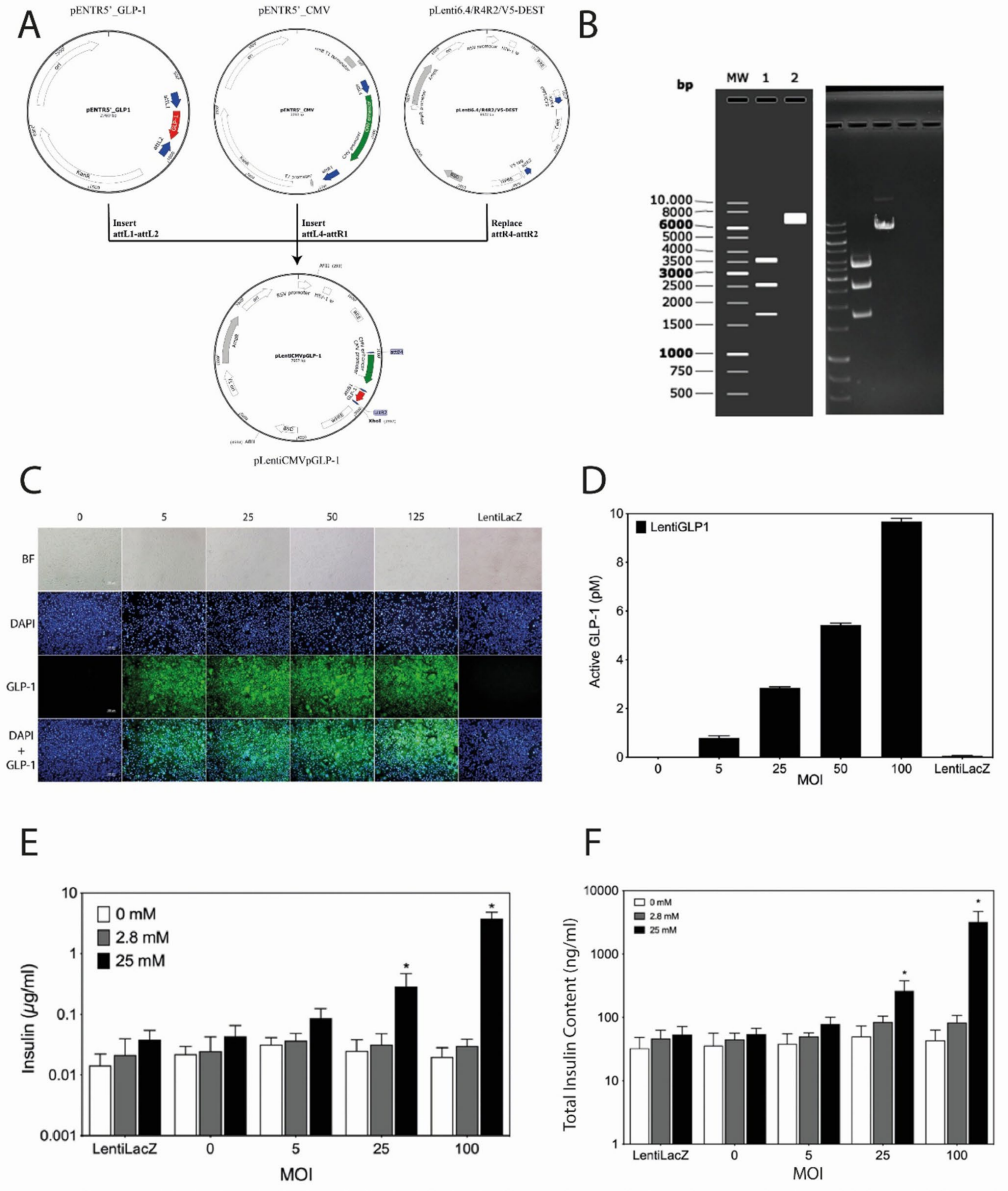
Construction, expression, and characterization of the third-generation HIV-based lentiviral vectors encoding the native GLP-1 protein. **(A)** Schematic representation of the lentiviral expression plasmid construction using Multisite Gateway^®^ LR recombination. The entry vector containing the *GLP-1* coding sequence (pENTR5’_GLP-1), flanked by attL1 and attL2 recombination sites, and an additional entry vector containing the CMV promoter (pENTR5’_CMV), flanked by attL4 and attR1 sites, were recombined with the destination vector pLenti6.4/R4R2/V5-DEST™, which contains attR4 and attR2 sites, using LR Clonase enzyme mix. The resulting expression plasmid (pLentiCMVpGLP-1) includes the 193 bp length *GLP-1* gene under a CMV promoter along with lentiviral vector backbone sequences. The diagram was generated using SnapGene^®^ software. **(B)** Agarose gel electrophoresis analysis of pLentiCMVpGLP-1. The left panel illustrates the expected fragment pattern, while the right panel shows the actual gel results. Well 1 contains the CMV promoter-driven GLP-1 expression plasmid digested with AflII and XhoI, and well 2 contains the undigested plasmid. (1% agarose gel; MW: GeneRuler™ 1 kb Ladder) **(C)** Immunofluorescence analysis of GLP-1 protein (green) expression in HepG2 cells transduced with LentiGLP-1 at varying multiplicities of infection (MOI: 0, 5, 25, 50, 125). Nuclei were counterstained with DAPI (blue). LentiLacZ-transduced cells (MOI:125) served as a control. Scale bars: 100 μm. Magnification: 20X. BF: Bright field **(D)** ELISA quantification of active GLP-1 levels in supernatants from HepG2 cells transduced with LentiGLP-1 (MOI: 0, 5, 25, 50, 100). LentiLacZ (MOI: 125) was used as a control. Statistical analysis was performed using one-way ANOVA. (*n* = 6/group, *p* < 0,0001) **E-F.** Evaluation of the effects of LentiGLP-1 transduction at varying MOI (0, 5, 25, 100) on insulin synthesis and secretion in the MIN6 pancreatic beta cell line. LentiLacZ at 100 MOI was used as a control. Statistical analysis was performed using two-way ANOVA. (*n* = 5/group; *p < 0.0001)

The entry vector encoding the bioactive GLP-1(7–37) fragment was constructed as described in our previous study [13]. After the successful generation of the GLP-1-containing entry vector, the expression construct was assembled using the Multisite Gateway system. During the LR reaction, the attL1 and attL2 sites of the GLP-1 entry vector (pENTR5’_GLP-1) and the attL4 and attR1 sites of the promoter entry vector (pENTR5’_CMV) were recombined with the attR4 and attR2 sites of the destination vector (pLenti6.4/R4R2/V5-DEST), via the Gateway^®^ LR Clonase™ II Enzyme Mix (Fig. 1A). Plasmid recombination and insert orientation were verified by AflII/XhoI restriction digestion, which was expected to yield three fragments of 3656 bp, 2574 bp, and 1727 bp (total 7957 bp). Agarose gel electrophoresis confirmed the presence of fragments of the expected sizes, thereby validating the correct insertion and orientation of the *GLP-1* gene within the expression vector (Fig. 1B). Following confirmation of orientation by restriction mapping, the plasmids were sequence-verified using Sanger sequencing, which confirmed the integrity and accuracy of the *GLP-1* coding sequence, with no detected mutations (data not shown).

### Functional analysis of LentiGLP-1

Subsequent to the successful construction of the GLP-1–encoding expression plasmid (pLentiCMVpGLP-1), third-generation lentiviral particles were produced via co-transfection with the packaging plasmids encoding Gag-Pol, Rev, and the VSV-G envelope protein. Quantification by qPCR indicated an average functional titer of approximately 1 × 10¹⁰ TU/mL. To assess the functional expression of GLP-1, HepG2 cells were transduced with LentiGLP-1. Immunofluorescence staining performed 72 h post-transduction using a GLP-1-specific antibody revealed intracellular green fluorescence signals indicative of GLP-1 protein expression. A concentration-dependent increase in fluorescence intensity was observed with increasing multiple of injection (MOI) values. Notably, no fluorescence was detected in HepG2 cells transduced with the control vector LentiLacZ at MOI 125 (Fig. 1C). GLP-1 peptide synthesis and secretion were further confirmed by ELISA. Supernatants from LentiGLP-1-transduced HepG2 cells displayed a significant and concentration-dependent increase in active GLP-1 levels, whereas no GLP-1 was detected in LentiLacZ controls (Fig. 1D). Furthermore, the functional activity of LentiGLP-1 was assessed by a GSIS assay. GLP-1 expression significantly enhanced insulin secretion under high-glucose conditions (25 mM), with no significant effect observed under low-glucose conditions. This insulinotropic response was particularly evident at MOI levels of 25 and 100, while MIN6 cells transduced with the LentiLacZ control vector showed no increase in insulin release (Fig. 1E). Furthermore, when both extracellular and intracellular insulin contents were considered, a significant increase in total insulin levels was observed at the same MOI concentrations (Fig. 1F). These findings demonstrate that the LentiGLP-1 vector mediates the production of functionally active GLP-1, confirming its biological activity in vitro.

### Therapeutic efficacy of LentiGLP-1 vector in STZ-induced newborn rat model

**Fig. 2:**
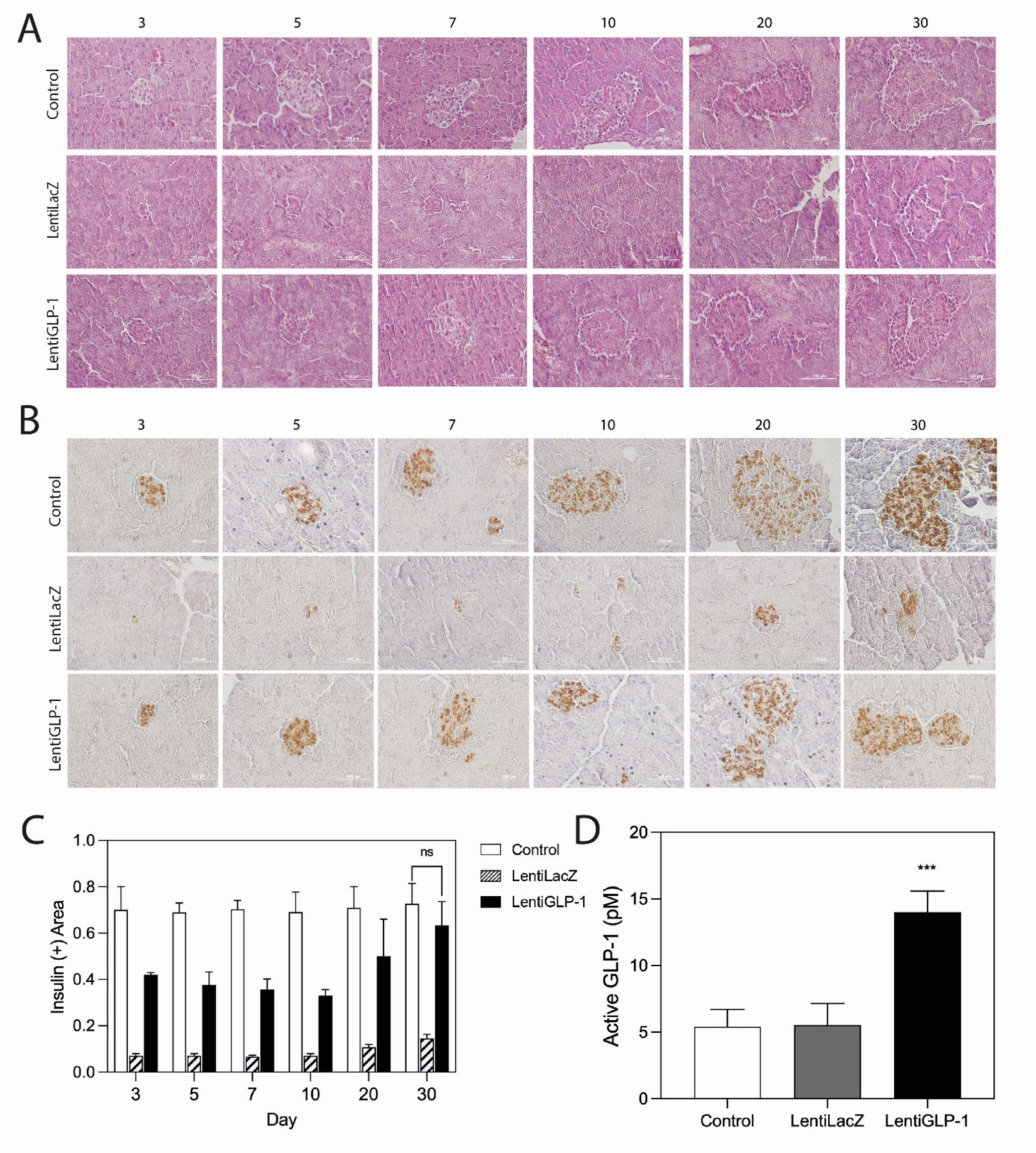
Histological and biochemical analysis of pancreatic tissues and serum GLP-1 levels in neonatal rats following STZ and lentiviral vector administration. **(A)** H&E staining of pancreatic sections from neonatal rats sacrificed at various time points. The control group consisted of healthy animals not subjected to STZ treatment. Experimental groups received STZ 48 h after birth, followed by either LentiGLP-1 or LentiLacZ injection at 72 h postnatally. The numbers above each image indicate the number of days post-lentiviral vector injection at which the tissue was collected. Representative images were acquired at 64X magnification. Scale bars: 100 μm. (*n* = 3/group) **(B)** Immunohistochemical staining for insulin in pancreatic sections from neonatal rats sacrificed at the indicated time points. Representative images were captured at 64X magnification. (*n* = 3/group) **(C)** Beta cell area scores of neonatal rats in the control, LentiLacZ, and LentiGLP-1 groups at various time points. Each pancreatic section was imaged using an Olympus IX81 motorized inverted fluorescence microscope, and insulin-positive areas were quantified using ImageJ software. Data were analyzed by two-way ANOVA, revealing statistically significant differences for group (*p* < 0.0001), time (*p* < 0.0001), and group × time interaction (*p* = 0.0006). Scale bars: 100 μm. (*n* = 3/group) **(D)** Comparative quantification of active GLP-1 concentrations in the blood serum of neonatal rats from the control, LentiLacZ, and LentiGLP-1 groups, sacrificed on day 20 post-lentiviral vector injection. To prevent enzymatic degradation of GLP-1, serum samples were treated with a DPP-4 inhibitor. Active GLP-1 levels were measured via ELISA and confirmed through fluorometric detection. Statistical evaluation was conducted using one-way ANOVA followed by Dunnett’s post hoc test, revealing significant differences among groups (***p = 0.0006; *n* = 3/group)

Following in vitro validation, the in vivo therapeutic efficacy of LentiGLP-1 was assessed in the STZ-induced neonatal diabetic rat model to evaluate its effects on hyperglycemia, β-cell differentiation, and potential extra-pancreatic outcomes. Accordingly, neonatal SD rats were assigned to three experimental groups: two groups received a single STZ administration, followed 24 h later by injection of either LentiGLP-1 or the control vector LentiLacZ, while a third group served as healthy controls receiving PBS. Histological analysis of pancreatic sections stained with H&E showed a significant increase in islet volume in healthy controls during the early neonatal period (days 3–10), with islets resembling adult-like morphology by day 20. On the other hand, STZ-treated animals receiving LentiLacZ exhibited smaller islets, indicative of extensive β-cell loss secondary to STZ-mediated cytotoxicity. Importantly, LentiGLP-1-treated animals showed a steady increase in islet volume from day 3 through day 30, reflecting a similar developmental pattern as the healthy controls (Fig. 2A). Immunohistochemical staining for insulin revealed strong and consistent insulin expression in control animals at all examined time points. The LentiLacZ group showed reduced insulin-positive staining at early time points (days 3, 5, 7, and 10), with only weak and limited insulin expression detectable by day 20, reflecting limited regenerative ability. Conversely, the LentiGLP-1-treated group demonstrated significantly enhanced insulin expression starting from day 3, which was maintained through day 30 (Fig. 2B). Quantitative analysis of insulin-positive areas confirmed qualitative observations, revealing a statistically significant reduction in insulin-positive areas in both LentiLacZ- and LentiGLP-1-treated groups immediately after STZ administration. However, a time-dependent recovery and expansion of β-cell area was observed only in the LentiGLP-1 group. At days 20 and 30, insulin-positive areas in the LentiGLP-1-treated animals were significantly higher than at earlier time points and greater than those observed in the LentiLacZ group (Fig. 2C). Consistent with these histological findings, analysis of blood samples by ELISA demonstrated that LentiGLP-1 injection in neonatal rats elicited a threefold increase in circulating active GLP-1, compared to control and LentiLacZ-treated groups (Fig. 2D).

### Investigation of the effects of GLP-1 on pancreatic cell differentiation and regeneration in neonatal rats

**Fig. 3:**
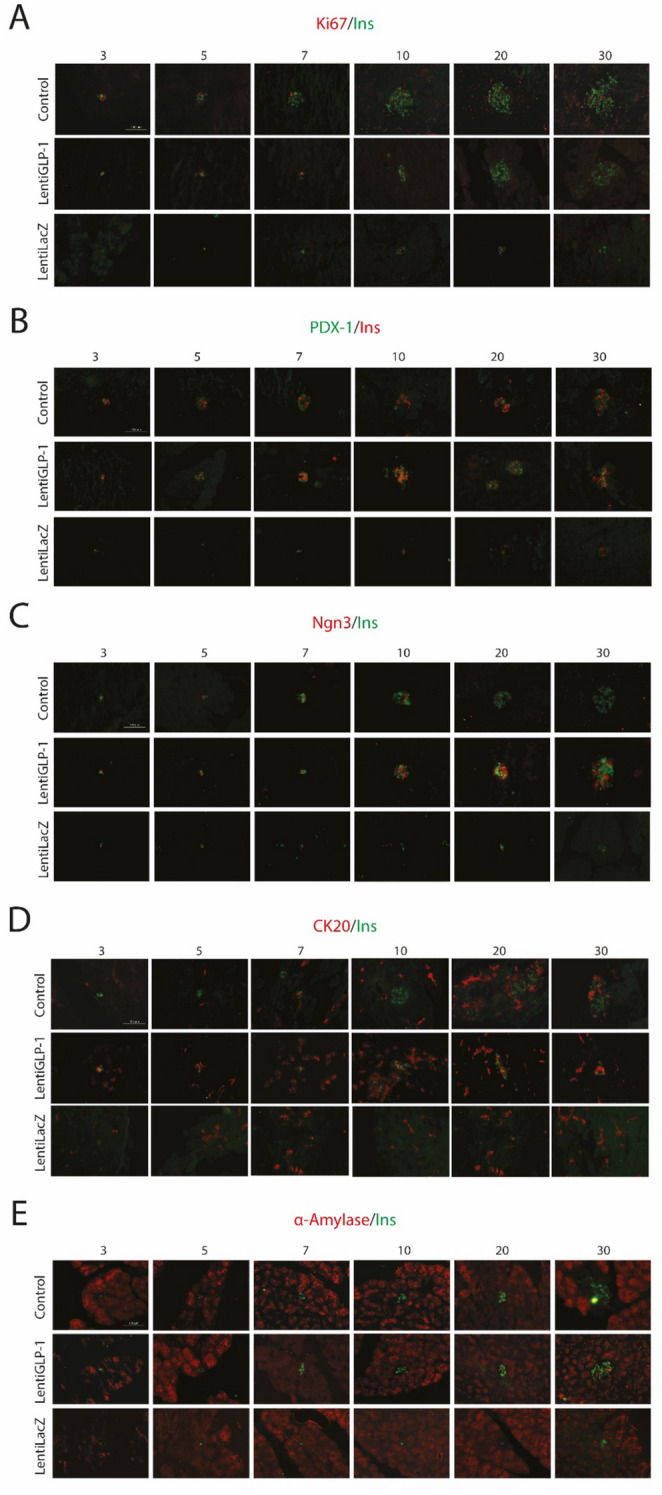
Dual immunofluorescence staining of pancreatic sections from neonatal rats to evaluate the origin of insulin-positive cells following STZ and lentiviral vector administration. **(A)** Dual immunofluorescence staining for insulin (green) and Ki67 (red) in pancreatic sections from neonatal rats in the control, LentiLacZ, and LentiGLP-1 groups. The control group includes healthy animals that did not receive STZ. In experimental groups, STZ was administered 48 h postnatally, followed by i.p. injection of either LentiGLP-1 or LentiLacZ at 72 h after birth. The time points indicated above each panel represent days post-lentiviral vector injection at the time of sacrifice. Images were acquired using a 64X objective lens. Scale bars: 100 μm. (*n* = 3/ group) **(B)** Dual immunofluorescence staining for PDX-1 (green) and insulin (red) in pancreatic sections. Images were obtained using a 64X magnification. Scale bars: 100 μm. (*n* = 3/group) **(C)** Dual immunofluorescence staining for Ngn3 (red) and insulin (green) in pancreatic sections. Magnification: 64X. Scale bars: 100 μm. (*n* = 3/group) **(D)** Dual immunofluorescence staining for cytokeratin 20 (CK20, red) and insulin (green) in pancreatic sections. Magnification: 64X. Scale bars: 100 μm. (*n* = 3/group) **(E)** Dual immunofluorescence staining for pancreatic alpha-amylase (red) and insulin (green) in pancreatic sections. Images were captured at 64X magnification. Scale bars: 100 μm. (*n* = 3/group)

To further elucidate the role of GLP-1 in pancreatic development, dual immunofluorescence staining was performed on neonatal pancreatic tissues to monitor the progression of pancreatic maturation during the neonatal period, determine the origins of insulin-positive cells, and investigate the specific effects of GLP-1 expression. Insulin/Ki67 co-staining revealed ongoing β-cell proliferation in control animals throughout the developmental period, whereas STZ-treated rats receiving the control vector LentiLacZ displayed minimal β-cell proliferation. By contrast, LentiGLP-1-treated rats exhibited pronounced insulin/Ki67 co-localization, indicating that GLP-1 expression enhances β-cell proliferation post STZ-induced damage (Fig. 3A). Dual immunofluorescence staining for insulin and PDX-1, a transcription factor critical for β-cell identity, showed strong co-expression in the LentiGLP-1 group as early as postnatal day 7 and persisting through later time points, suggesting promotion of functional β-cell regeneration. Control animals displayed normal insulin/PDX-1 co-localization consistent with physiological development, while the LentiLacZ group exhibited sparse insulin-positive cells with minimal PDX-1 expression (Fig. 3B). Analysis of insulin/Ngn3 co-staining, a marker of endocrine progenitors, indicated that GLP-1 promotes β-cell neogenesis by enhancing differentiation of Ngn3-positive progenitors into insulin-expressing cells, an effect absent in the LentiLacZ group, whereas control animals showed mild Ngn3 expression persisting until at least postnatal day 20 (Fig. 3C). Similarly, insulin/CK20 staining demonstrated that LentiGLP-1 induces transdifferentiation of ductal cells into insulin-positive β-cells after STZ-mediated β-cell depletion, while no such co-localization was observed in control or LentiLacZ-treated animals further supporting the specificity of the GLP-1-mediated response (Fig. 3D). Insulin/alpha-amylase staining revealed no evidence of acinar-to-beta cell conversion in any group (Fig. 3E). Collectively, these results indicate that LentiGLP-1 enhances β-cell proliferation, neogenesis, and ductal-to-beta cell transdifferentiation, whereas the control and LentiLacZ groups show limited regenerative capacity.

### Therapeutic efficacy of LentiGLP-1 vector in adult OD rat model

T2DM is a metabolic disease characterized by insulin resistance and progressive β-cell dysfunction. To elucidate the long-term effects of sustained GLP-1 expression on pancreatic biology, an adult OD rat model was employed to examine its influence on both endocrine and exocrine compartments. Six-week-old male SD rats were divided into four groups. The control group received a standard diet, while three experimental groups were fed a high-fat diet to induce obesity. After obesity induction, one high-fat group (‘Obese’ control) received PBS, and the other two were given a single i.p. dose of STZ (40 mg/kg) to induce hyperglycemia. Rats with blood glucose ≥ 250 mg/dL were classified as diabetic. Seven days after STZ administration, diabetic animals were treated with either LentiGLP-1 or LentiLacZ, and blood glucose and body weight were monitored weekly for four weeks post-treatment.

**Fig. 4:**
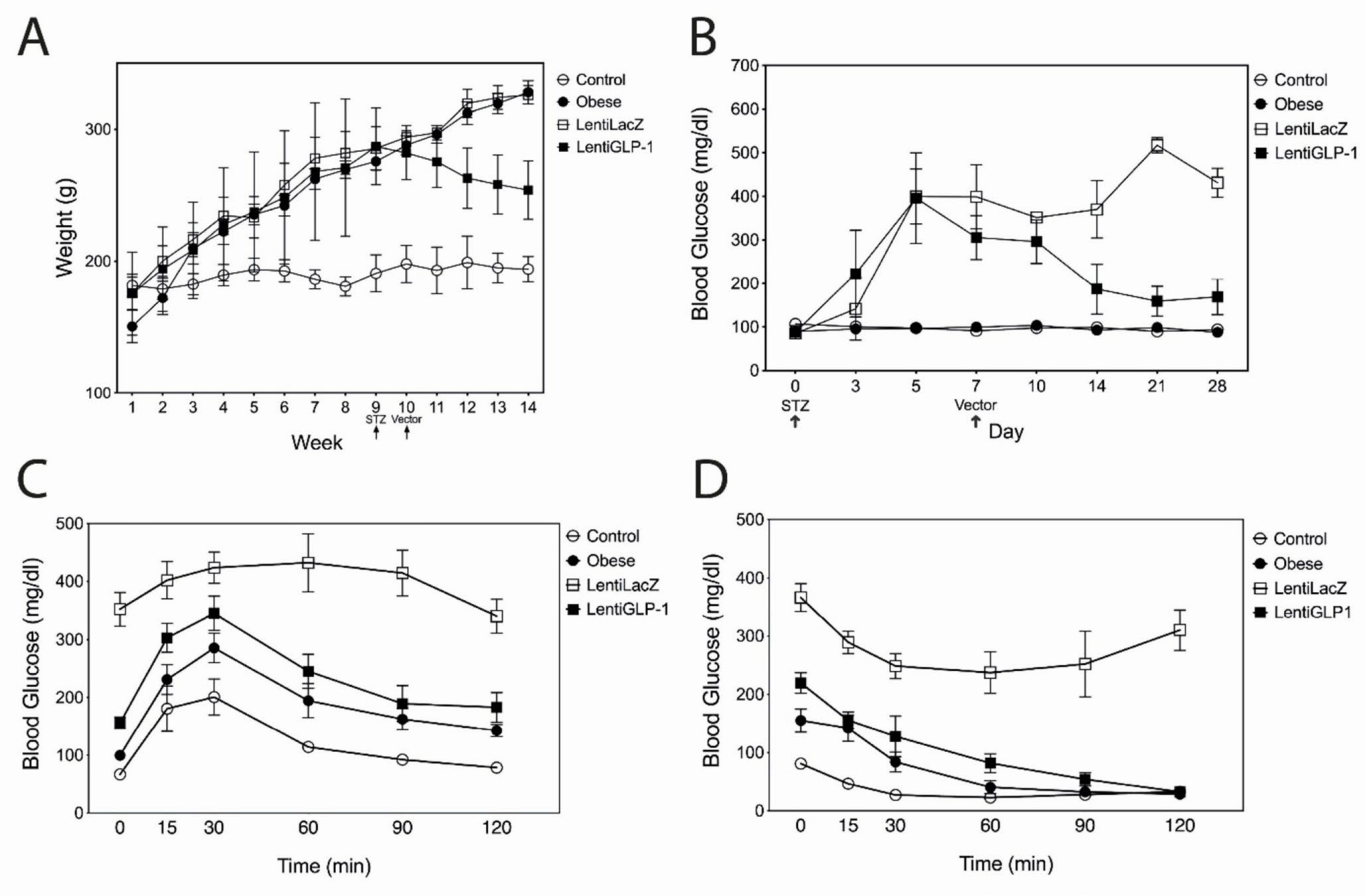
Metabolic assessments in healthy and obese diabetic SD rats. **(A)** Time-course analysis of body weight. Control SD rats were maintained on a standard diet, while obese SD rats received a high-fat diet; neither group underwent STZ treatment. Experimental groups (LentiLacZ and LentiGLP-1) were also fed a high-fat diet and received STZ administration in week 9. SD rats with blood glucose levels ≥ 250 mg/dL on days 3 and 5 post-STZ injections were considered diabetic. Lentiviral vector delivery was performed in week 10. (Statistical analysis: Two-Way ANOVA; *p* < 0.0001, *n* = 8/group) **(B)** Time-dependent changes in blood glucose levels in SD rat groups. Glucose levels were assessed on days 0, 3, 5, 7, 10, 14, 21, and 28 post-STZ injection. (Statistical analysis: Two-way ANOVA; *p* < 0.0001, *n* = 8/group) **(C)** IPGTT profiles of SD rat groups 3 weeks after gene delivery. The test was performed following an 8-hour fasting period, with i.p. administration of glucose at a dose of 2 g/kg. Blood glucose levels were measured at 0 (basal), 15, 30, 60, 90, and 120 min after glucose injection. (Statistical analysis: Two-way ANOVA; *p* = 0.0259, *n* = 8/group) **(D)** ITT profiles of Control, Obese, LentiLacZ, and LentiGLP-1 SD rat groups 3 weeks after gene delivery. Following a 7-hour fasting period, SD rats received an i.p. injection of Humalog insulin at a dose of 1 U/kg. Blood glucose levels were measured at 0 (basal), 15, 30, 60, 90, and 120 min post-insulin injection. (Statistical analysis: Two-way ANOVA; *p* = 0.0052, *n* = 8/group)

Control rats exhibited minimal weight gain throughout the study, whereas high-fat feeding resulted in progressive obesity, becoming significant from week 4. Among diabetic groups, LentiLacZ injection did not alter body weight relative to untreated obese rats. In contrast, LentiGLP-1 treatment elicited a significant and sustained reduction in body weight beginning at week 10 (Fig. 4A). Blood glucose analyses demonstrated that control and obese non-diabetic rats maintained normoglycemia (~ 100 mg/dL). STZ administration induced marked hyperglycemia, which persisted in the LentiLacZ group (350–550 mg/dL) but was progressively reduced by LentiGLP-1 treatment, lowering blood glucose levels below the diabetic threshold (< 250 mg/dL) by day 14 (Fig. 4B). The impact of LentiGLP-1 on glucose homeostasis and insulin sensitivity was further evaluated using metabolic tests. IPGTT revealed severe glucose intolerance in obese and LentiLacZ-treated diabetic rats, with glucose levels exceeding 300 mg/dL. By contrast, LentiGLP-1-treated rats demonstrated significantly improved glucose disposal, with glucose levels declining below 250 mg/dL by 60 min (Fig. 4C). Similarly, ITT indicated preserved insulin responsiveness in controls but markedly reduced sensitivity in obese and LentiLacZ-treated diabetic rats, where glucose levels remained > 250 mg/dL. LentiGLP-1 injection significantly enhanced insulin sensitivity, producing a pronounced glucose-lowering response from 30 min onward and approaching control values by 60 min (Fig. 4D).

**Fig. 5:**
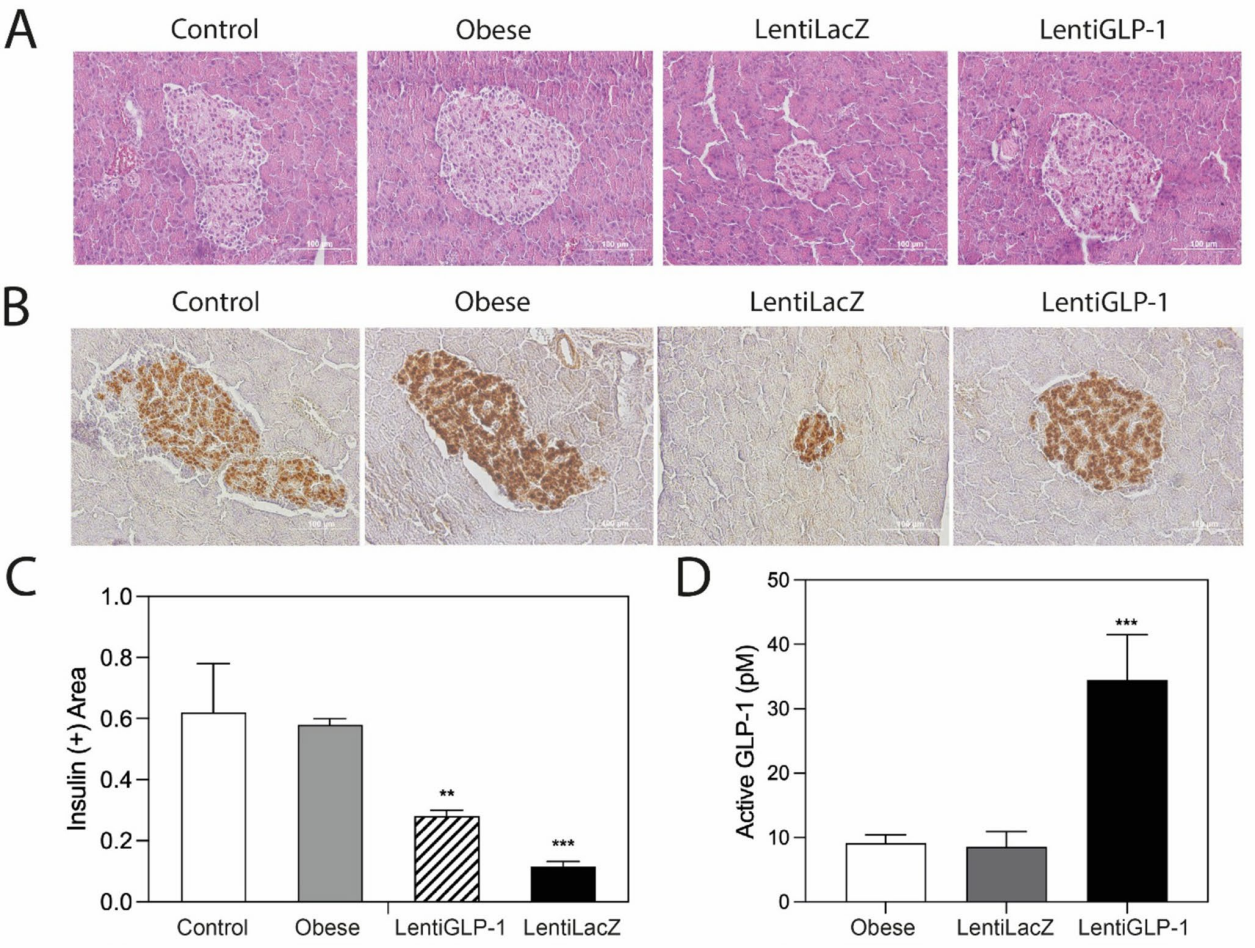
Histological and biochemical evaluation of pancreatic tissue and serum GLP-1 levels in adult SD rats following STZ and lentiviral vector administration. **(A)** H&E staining of pancreatic sections. Control rats were maintained on a standard diet, and obese rats were fed a high-fat diet; neither group received STZ treatment. Experimental groups (LentiLacZ and LentiGLP-1) were fed a high-fat diet, administered STZ, and subsequently received lentiviral vectors. Representative images are shown at 40X magnification. Scale bars: 100 μm. (*n* = 8/group) **(B)** Immunohistochemical staining for insulin in pancreatic sections. Representative images were captured at 40X magnification. Scale bars: 100 μm. (*n* = 8/group) **(C)** Quantification of β-cell area. Insulin-positive areas were analyzed using ImageJ software on images obtained with an Olympus IX81 motorized inverted fluorescence microscope. Data were statistically evaluated using one-way ANOVA. (*p* = 0.0002; *n* = 8/group) **(D)** Comparative quantification of active GLP-1 concentrations in the blood serum of adult SD rats. Blood samples were treated with a DPP-4 inhibitor to prevent degradation, and active GLP-1 levels were measured by ELISA with fluorometric confirmation. Statistical analysis was performed using one-way ANOVA followed by Dunnett’s test (***p < 0.0001; *n* = 8/group)

One month after STZ administration, animals were sacrificed, and blood and abdominal organs were collected. Histological analysis of pancreatic sections stained with H&E revealed that the overall pancreatic architecture and islet morphology in control and obese groups were largely preserved, with clearly distinguishable Langerhans islets showing similar organization. Conversely, STZ-treated diabetic rats administered LentiLacZ exhibited noticeably smaller islets. Notably, islets from LentiGLP-1-treated rats appeared larger compared to those in the LentiLacZ group (Fig. 5A). Immunohistochemical staining for insulin revealed that insulin immunoreactivity was strong and widespread in both control and obese rats. However, due to the β-cell-specific cytotoxicity of STZ, Langerhans islets from LentiLacZ-treated diabetic rats were markedly reduced in size, accompanied by a pronounced decrease in insulin-positive cells. On the other hand, LentiGLP-1-treated diabetic rats exhibited partial preservation of β-cell mass, with a noticeably higher number of insulin-positive cells compared to the LentiLacZ group (Fig. 5B). Quantitative analysis of insulin-positive areas showed no significant difference in the proportion of insulin-positive areas between the control and obese groups. However, LentiGLP-1-treated rats displayed a markedly greater percentage of insulin-positive area compared to LentiLacZ-treated diabetic rats, supporting the β-cell–preserving effect of GLP-1 expression (Fig. 5C). Consistent with these metabolic outcomes, ELISA analysis of serum samples demonstrated that LentiGLP-1 injection in OD rats resulted in a threefold increase in circulating active GLP-1 relative to obese and LentiLacZ-treated groups (Fig. 5D). These findings together demonstrate that sustained GLP-1 expression confers β-cell protection, ameliorates hyperglycemia, improves glucose tolerance, enhances insulin sensitivity, and favorably regulates body weight in an adult OD rat model, highlighting its therapeutic potential for T2DM.

### Dual immunofluorescence analysis of insulin-positive cell origins and pathways of β-cell regeneration in adult rats

**Fig. 6:**
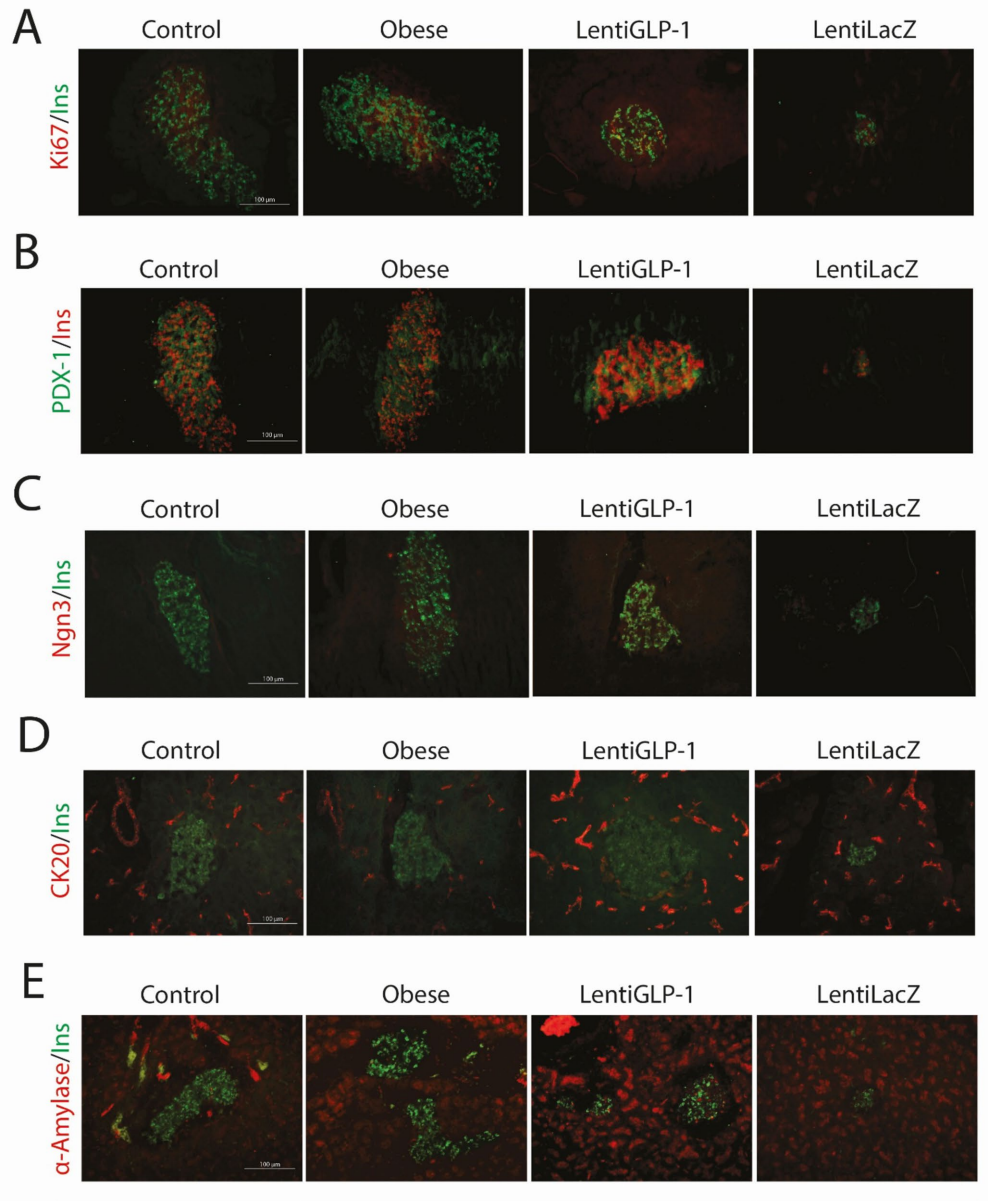
Dual immunofluorescence analysis of pancreatic sections to investigate the origin and regeneration of insulin-positive cells in adult SD rats following STZ-induced injury and lentiviral vector injection. **(A)** Dual immunofluorescence staining for insulin (green) and Ki67 (red) to evaluate β-cell proliferation. Control SD rats were maintained on a standard diet, while obese SD rats received a high-fat diet; neither group was treated with STZ. LentiLacZ and LentiGLP-1 groups were fed a high-fat diet, treated with STZ, and subsequently received lentiviral vectors. Ki67 expression indicates proliferative activity, and insulin staining marks insulin-positive cells. Images were acquired using 40X objective lens. Scale bars: 100 μm. (*n* = 8/group) **(B)** Dual immunofluorescence staining for PDX-1 (green) and insulin (red) to assess expression of the pancreatic transcription factor PDX-1 in insulin-positive cells. Images captured at 40X magnification. Scale bars: 100 μm. (*n* = 8/group) **(C)** Dual immunofluorescence staining for Ngn3 (red) and insulin (green). Magnification: 40X. Scale bars: 100 μm. (*n* = 8/group) **(D)** Dual immunofluorescence staining for CK20 (red) and insulin (green). Magnification: 40X. Scale bars: 100 μm. (*n* = 8/group) **(E)** Dual immunofluorescence staining for pancreatic alpha-amylase (red) and insulin (green). Images were captured at 40X magnification. Scale bars: 100 μm. (*n* = 8/group)

Following immunohistochemical analyses demonstrating the effects of GLP-1 on β-cells in OD adult rats, dual immunofluorescence staining was performed to investigate the cellular origins and mechanisms underlying insulin-positive cell formation, focusing on proliferation, differentiation, and neogenesis. Insulin/Ki67 co-staining revealed low basal β-cell proliferation in control animals, whereas obese rats showed increased co-localization, consistent with compensatory proliferation in response to higher insulin demand. LentiLacZ-treated diabetic rats exhibited minimal insulin/Ki67 co-localization, reflecting reduced β-cell proliferation and smaller islet size. In contrast, LentiGLP-1-treated rats displayed pronounced insulin/Ki67 co-localization, with strong nuclear Ki67 expression in insulin-positive cells, suggesting that GLP-1 enhances β-cell replication (Fig. 6A). Dual immunofluorescence staining for insulin and PDX-1 demonstrated that control and obese animals maintained β-cell identity, whereas LentiLacZ-treated rats showed markedly reduced PDX-1/insulin co-expression, indicative of β-cell loss. In the LentiGLP-1 group, strong nuclear PDX-1 expression in insulin-positive cells suggested improved β-cell maintenance and possible generation of new insulin-producing cells (Fig. 6B). Analysis of insulin/Ngn3 co-staining revealed minimal neogenesis in control and obese animals, while co-localization in the LentiGLP-1 group indicated that GLP-1 reactivates Ngn3 expression and promotes differentiation of progenitor cells into insulin-producing cells, an effect absent in the LentiLacZ group, confirming this neogenic effect is specifically mediated by GLP-1 (Fig. 6C). Similarly, insulin/CK20 co-staining showed no ductal-to-β-cell conversion in control, obese, or LentiLacZ-treated animals, indicating that ductal-to-endocrine conversion is rare in the adult pancreas. In contrast, LentiGLP-1-treated rats exhibited CK20/insulin co-localization in peri-islet regions, suggesting an early or intermediate stage of differentiation of ductal epithelial cells into insulin-producing β-cells (Fig. 6D). Insulin/α-amylase staining revealed no acinar-to-β-cell conversion in any group, confirming that acinar cells do not contribute to β-cell regeneration in the adult pancreas under either physiological or GLP-1–induced conditions (Fig. 6E). Altogether, these findings indicate that LentiGLP-1 enhances β-cell proliferation, maintenance, and regeneration through neogenesis and ductal-to-β-cell transdifferentiation, whereas control, obese, and LentiLacZ-treated animals display limited regenerative capacity.

### Effects of LentiGLP-1 gene transfer on the liver in adult and neonatal rats

**Fig. 7:**
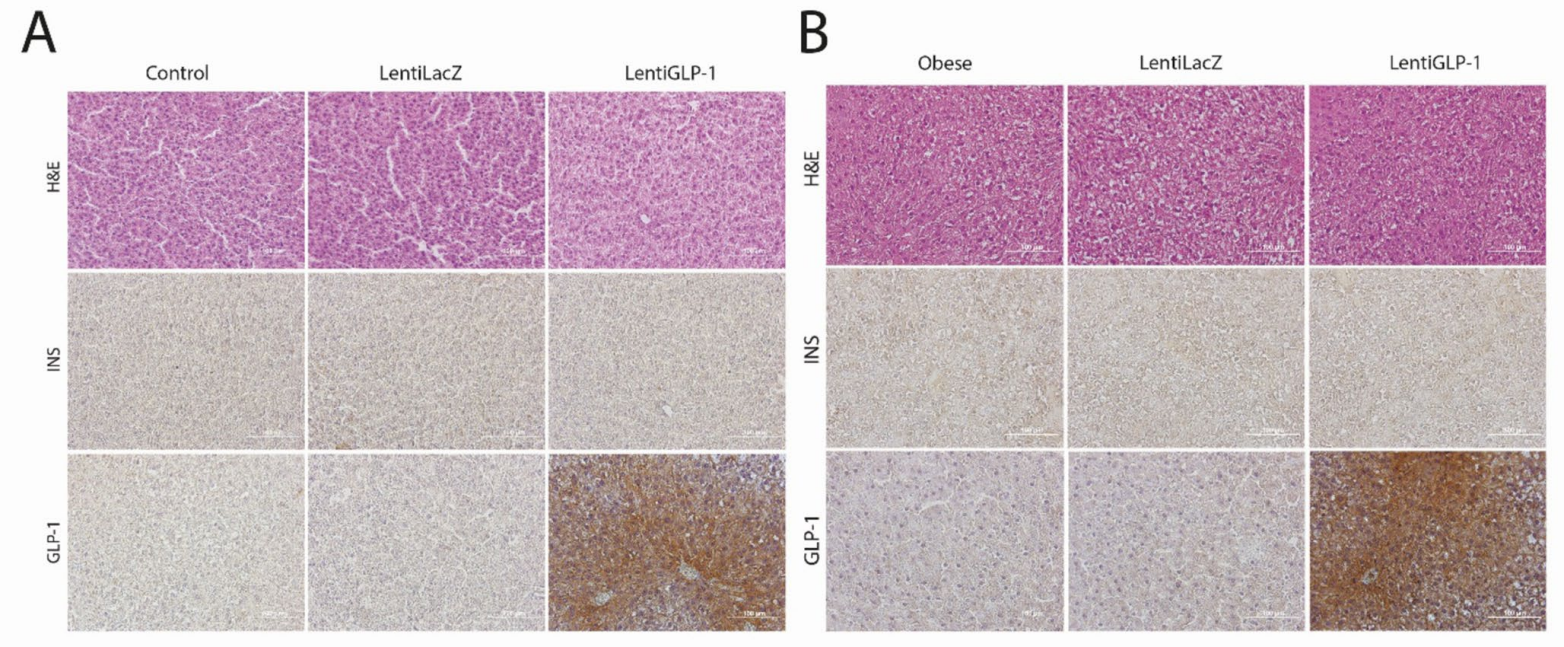
Immunohistochemical analysis of hepatic sections from neonatal and adult SD rats to assess the effects of GLP-1 gene transfer on hepatic insulin and GLP-1 expressions. **(A)** H&E, insulin (INS), and GLP-1 immunohistochemical staining of liver sections from neonatal SD rats in the Control, LentiLacZ, and LentiGLP-1 groups, sacrificed on day 20 post-lentiviral vector injection. The control group consisted of healthy animals that did not receive STZ or lentiviral vector administration. Images were acquired at 64X magnification. Scale bars: 100 μm. (*n* = 3/group) **(B)** H&E, insulin (INS), and GLP-1 immunohistochemical staining of liver sections from adult SD rats. The obese group was fed a high-fat diet and did not receive STZ administration. Images were captured at 40X magnification. Scale bars: 100 μm. (*n* = 8/group)

The liver, sharing endodermal origin and functional similarity with the pancreas, was examined in neonatal and adult rats as a secondary target organ to assess potential off-target effects. In neonatal rats (Fig. 7A), LentiGLP-1 injection induced robust hepatic GLP-1 expression, whereas control and LentiLacZ-treated groups exhibited no detectable signal. Insulin immunostaining was absent in all neonatal groups, indicating that hepatocytes did not acquire insulin-producing phenotypes. Consistently, in adult rats (Fig. 7B), LentiGLP-1 treatment elicited pronounced GLP-1 expression, while obese and LentiLacZ groups remained negative, and insulin positivity was undetectable in all adult livers.

## Discussion

GLP-1 is a potent incretin hormone that exerts multifaceted effects on pancreatic β-cells. Beyond enhancing glucose-dependent insulin secretion, GLP-1 promotes β-cell survival, increases replication, suppresses intracellular stress factors, and triggers regenerative mechanisms [15]. Moreover, GLP-1 can stimulate the differentiation of progenitor cells into β-cells by activating key transcription factors such as *Pdx-1* and *Ngn3* [16, 17]. It has also been reported to induce the transdifferentiation of exocrine cell types, including ductal or acinar cells, into insulin-producing β-cells [18]. These pleiotropic actions make GLP-1 an attractive therapeutic molecule for diabetes treatment. Consequently, pharmacological GLP-1 receptor agonists have been developed and are now widely used in clinical practice. However, many of these agonists are associated with adverse effects, including gastrointestinal discomfort, and require frequent (daily or weekly) injections to maintain consistent incretin levels and bioactivity [19, 20]. Additionally, the rapid degradation of endogenous GLP-1 in circulation limits its therapeutic efficacy. To overcome these limitations, we developed an approach to achieve sustained and long-term in vivo GLP-1 expression. For this purpose, third-generation LentiGLP-1 were employed to achieve stable *in vivo *expression of the native active form of GLP-1 (7–37). In vitro ELISA and GSIS assay demonstrated that LentiGLP-1 successfully produced biologically active GLP-1 and enhanced insulin secretion.

The potential impact of long-term GLP-1 expression on pancreatic development remains incompletely understood. To address this, a neonatal rat model, characterized by active pancreatic morphogenesis and high β-cell regenerative capacity, was subjected to STZ-induced β-cell injury, followed by injection of either LentiGLP-1 or control LentiLacZ vectors. This approach allowed us to examine whether sustained GLP-1 expression could support β-cell preservation, proliferation, and neogenesis during early postnatal development, a critical window when β-cell mass rapidly expands and the pancreas exhibits heightened plasticity. Histological analyses revealed that LentiGLP-1-treated neonatal rats exhibited a progressive increase in islet volume from postnatal day 3 through day 30, closely mirroring the developmental trajectory of healthy controls, whereas STZ-treated LentiLacZ animals displayed persistent islet atrophy, reflecting the high susceptibility of neonatal β-cells to cytotoxic injury [21]. Immunohistochemical evaluation demonstrated markedly increased insulin-positive areas in LentiGLP-1-treated neonatal rats, indicative of both β-cell preservation and activation of neogenic pathways. Serum analysis confirmed functional GLP-1 expression, with circulating levels approximately threefold higher than in controls, supporting the conclusion that the observed enhancements in insulin immunoreactivity and islet area were directly mediated by LentiGLP-1 treatment. These results provide robust in vivo evidence of GLP-1’s regenerative potential in neonatal rats, offering mechanistic insight and reinforcing its therapeutic relevance for neonatal diabetes and conditions characterized by impaired β-cell mass.

Double immunofluorescence staining with Ki67 was performed to simultaneously assess β-cell identity and proliferation during neonatal pancreatic development. This approach enabled precise evaluation of β-cell regenerative activity. In control neonatal rats, Ki67/insulin co-localization indicated active β-cell proliferation and expansion of the β-cell mass, reflecting normal postnatal pancreatic growth. In contrast, the absence of Ki67/insulin co-staining in STZ-treated LentiLacZ rats suggested an inadequate proliferative response to injury, highlighting the vulnerability of neonatal β-cells to cytotoxic insult. Conversely, the presence of Ki67/insulin-positive cells in the LentiGLP-1 group demonstrated that sustained GLP-1 expression supported β-cell regeneration by stimulating cell cycle entry in surviving β-cells, compensating for STZ-induced cell loss. This observation is consistent with previous studies showing that GLP-1 and its analogs enhance β-cell proliferation in vivo and emphasizes the capacity of GLP-1 to reactivate intrinsic proliferative programs in developing β-cells [22, 23].

To further explore endocrine cell differentiation and neogenesis, double immunofluorescence analyses were performed using PDX-1 and Ngn3 markers. PDX-1 regulates pancreatic progenitor development and maintains β-cell identity, while Ngn3 governs the differentiation of endocrine progenitors into α-, β-, δ-, and PP-cells [24]. Together, they provide insights into both β-cell maintenance and *de novo* generation of β-cells. In control neonatal rats, PDX-1/insulin co-staining confirmed normal β-cell differentiation and maturation, whereas reduced PDX-1/insulin signals in the LentiLacZ group indicated STZ-induced β-cell depletion. Notably, the LentiGLP-1 group exhibited increased numbers of PDX-1/insulin-positive cells, suggesting that GLP-1 preserved existing β-cells and stimulated neogenesis by activating essential transcriptional factors. Similarly, in neonatal controls, Ngn3/insulin co-staining observed up to postnatal day 20 indicated ongoing β-cell neogenesis from progenitors. In opposition, minimal Ngn3/insulin signal in the LentiLacZ group reflected extensive β-cell loss and impaired regenerative activity. The increase of Ngn3/insulin-positive cells in the LentiGLP-1 group demonstrated that GLP-1 reactivated the dormant regenerative potential of endocrine progenitors and promoted neogenesis. These findings align with reports showing that GLP-1 analogs, such as liraglutide or exendin-4, enhance β-cell mass through both proliferation and neogenic mechanisms [9, 25, 26].

To investigate GLP-1’s effects on exocrine pancreatic cells and potential transdifferentiation, double immunofluorescence staining was performed using CK20 (ductal) and α-amylase (acinar) markers. During normal pancreatic development, endocrine cells arise from multipotent progenitors in the pancreatic epithelium of the foregut endoderm (from E9.5–10 in rodents), whereas ductal and acinar cells differentiate from the trunk and tip domains, respectively [27]. This sequential differentiation along distinct, lineage-restricted trajectories preserves cell identity. Consistent with this, CK20/insulin co-expression was absent in control neonatal rats, reflecting distinct lineage origins. Conversely, CK20/insulin co-localization in STZ-treated LentiGLP-1 animals indicates that GLP-1 can recruit ductal progenitors toward an insulin-producing phenotype. This finding suggests an additional mechanism for β-cell mass expansion beyond proliferation or endocrine progenitor differentiation. These results are consistent with reports showing that GLP-1 induces insulin expression in rat (ARIP) and human (PANC-1) pancreatic cell lines [11]. By contrast, α-amylase/insulin double staining yielded no co-localization in any neonatal group, indicating that acinar cells did not undergo transdifferentiation into β-cells. This observation is consistent with reports indicating that acinar cells exhibit limited plasticity and are largely refractory to GLP-1-induced transdifferentiation [28]. Altogether, these dual labeling results demonstrate that LentiGLP-1 mediates β-cell regeneration in neonatal rats through β-cell proliferation, endocrine progenitor differentiation, and ductal-to-endocrine transdifferentiation. However, it remains insufficient to drive acinar cell conversion into insulin-producing cells.

Building on these neonatal findings, we next examined the therapeutic potential of sustained GLP-1 expression in adult rats, where β-cell plasticity is more limited. Given the pathophysiological relevance of T2DM, characterized by insulin resistance and β-cell dysfunction, the adult OD rat model was employed to further elucidate GLP-1’s regenerative effects under diabetic conditions. In this model, rats were fed a high-fat diet to induce obesity, followed by STZ administration to induce β-cell loss.

LentiGLP-1 injection in adult OD rats significantly improved metabolic outcomes. In this study, LentiGLP-1-treated OD rats gained less weight compared to LentiLacZ-treated rats. This reduction in weight gain is likely mediated by reduced food intake and enhanced metabolic efficiency, consistent with the central and peripheral regulatory actions of GLP-1. The marked reduction in blood glucose from persistently hyperglycemic levels (~ 350–550 mg/dL) in diabetic controls to below 250 mg/dL within two weeks of LentiGLP-1 treatment indicates enhanced insulin secretion. This rapid normalization of glycemia likely reflects GLP-1–mediated restoration of residual β-cell function together with increased glucose uptake. Metabolic challenge tests confirmed these effects. LentiGLP-1-treated OD rats showed markedly improved glucose tolerance during IPGTT and enhanced insulin sensitivity during ITT, with glucose levels approaching control values within 60 min. These results indicate that LentiGLP-1 not only modulates body weight but also improves systemic glucose homeostasis by reversing insulin resistance and enhancing glucose tolerance.

Histological and immunohistochemical analyses corroborated the metabolic findings, confirming the protective effects of GLP-1 on pancreatic morphology. Pancreatic sections from LentiGLP-1-treated rats exhibited larger, more organized islets compared with the markedly atrophic islets observed in LentiLacZ-treated OD rats. Quantitative evaluation revealed a significantly greater insulin-positive area in the LentiGLP-1 group, indicative of partial preservation of β-cell mass and enhanced insulin immunoreactivity. These results reinforce the cytoprotective and potentially regenerative effects of GLP-1 signaling, which are likely mediated through the cAMP–PKA and PI3K–Akt pathways, known to promote β-cell survival and function [29]. Consistent with these histological outcomes, ELISA analysis confirmed approximately a threefold elevation in circulating active GLP-1 levels in treated rats, validating efficient vector-mediated expression and providing a mechanistic basis for the observed improvements in glycemic control and β-cell preservation.

The origin of insulin-positive cells in adult OD rats was examined using double immunofluorescence staining to elucidate the cellular mechanisms underlying β-cell maintenance and regeneration in adult rats in response to GLP-1 treatment. Ki67/insulin co-staining revealed low basal β-cell proliferation in control rats, with a compensatory increase in obese animals to meet higher insulin demand. This proliferative capacity was markedly reduced in LentiLacZ-treated diabetic rats, consistent with diabetes progression. In contrast, LentiGLP-1-treated rats exhibited increased insulin/Ki67 co-localization, suggesting GLP-1 reactivated β-cell proliferation, likely via cyclin D1–mediated pathways [30]. PDX-1/insulin co-staining was preserved in controls and obese rats but reduced in LentiLacZ diabetics, whereas LentiGLP-1 treatment increased PDX-1/insulin-positive cells, indicating support of existing β-cells and promotion of new β-cell formation. Similarly, Ngn3/insulin double-positive cells emerged only in the LentiGLP-1 group, suggesting GLP-1 can reactivate progenitor-like cells and induce β-cell neogenesis. These findings are consistent with previous reports showing that GLP-1 and its analogs enhance adult β-cell proliferation, activate cell cycle–related proteins such as cyclin D1, and support β-cell survival [31–33]. Moreover, exendin-4 administration for two weeks in diabetic (db/db) mice was reported to increase PDX-1 expression and promote β-cell differentiation [34]. It has also been demonstrated in both in vitro and in vivo models that Ngn3 can be reactivated in the adult pancreas and that GLP-1 can support this process, thereby promoting neogenesis [5, 17].

CK20/insulin co-expression in LentiGLP-1-treated diabetic rats indicated that GLP-1 facilitates ductal-to-β-cell conversion even in adult pancreatic tissue, highlighting the plasticity of ductal progenitors in response to regenerative cues. On the other hand, α-amylase/insulin co-staining remained absent, confirming that acinar cells remain terminally differentiated and unresponsive to GLP-1–induced transdifferentiation. This resistance is likely due to their developmental divergence from the endocrine lineage and low GLP-1R expression. These observations are consistent with previous reports showing that GLP-1 can promote the conversion of ductal cells to endocrine cells in the rat pancreatic ductal (ARIP) cell line [35]. Although GLP-1R is expressed at low levels in ductal cells, its expression can increase under regenerative or stress conditions, potentially allowing GLP-1 to influence ductal cell fate [36]. By contrast, acinar cells, which have developmentally diverged from the endocrine lineage and express GLP-1R at very low levels, appear refractory to direct GLP-1–mediated effects [37, 38]. Notably, acinar-to-β-cell conversion requires high-level co-expression of key transcription factors such as *Pdx-1*, *Ngn3*, and *MafA* via adenoviral vectors, and has been achieved mainly in immunosuppressed adult mouse models [39]. These results indicate that LentiGLP-1 primarily promotes β-cell proliferation and differentiation from progenitor or ductal sources rather than acinar-to-endocrine conversion.

Following the evaluation of GLP-1 effects on the pancreas, the liver was examined in both models due to the shared developmental origin and metabolic interplay between these organs. Both liver and pancreas parenchyma derive from the endoderm, with stromal components from the mesoderm [40], highlighting structural and functional proximity that could theoretically allow similar cellular responses to LentiGLP-1. In both neonatal and adult rats, hepatic GLP-1 immunostaining was absent in control and LentiLacZ-treated animals but markedly increased in LentiGLP-1-treated rats, reflecting successful vector-mediated transgene expression. Notably, despite the high plasticity of neonatal hepatocytes, insulin immunostaining remained undetectable across all groups, indicating that GLP-1 did not induce transdifferentiation of hepatocytes into insulin-producing cells. These findings demonstrate that while LentiGLP-1 effectively drives GLP-1 expression in the liver, its pro-β-cell effects are pancreas-specific and do not extend to hepatocytes, suggesting organ-restricted responsiveness to GLP-1 despite shared embryonic origin.

These findings highlight the translational potential of LentiGLP-1 therapy across developmental stages. In neonatal rats, sustained GLP-1 expression robustly preserved and expanded β-cell mass, promoting proliferation and neogenesis during critical windows of pancreatic development, which may prevent or delay diabetes onset. In adult rats, where β-cell plasticity is limited, LentiGLP-1 partially restored β-cell mass and function, improving glucose homeostasis, insulin sensitivity, and body weight, likely via combined effects on β-cell proliferation, progenitor activation, and peripheral metabolism.

The lentiviral vector-mediated delivery employed in this study provides long-term, single-dose GLP-1 expression, overcoming the transient effects and repeated dosing requirements of conventional GLP-1R agonists. Sustained expression may improve therapeutic efficacy, stabilize glycemic control, and reduce treatment burden, supporting its potential for long-term diabetes management.

Future investigations should focus on comprehensive preclinical safety assessment, optimization of vector dosage, and evaluation of translational feasibility in humans, with particular attention to age-dependent responses and regenerative capacity. Moreover, elucidating the interactions between GLP-1–mediated β-cell regeneration and systemic metabolic pathways, as well as potential immunological or off-target effects of prolonged viral expression, will be critical to advancing clinical application. Notably, the origin of newly formed β-cells differs between developmental stages. In the neonatal pancreas, they arise mainly from progenitor and ductal cells, whereas in the adult pancreas regeneration occurs predominantly through replication of existing β-cells with only limited neogenesis. Collectively, these data provide compelling evidence that GLP-1–based regenerative therapy can simultaneously preserve β-cell function and enhance systemic metabolic homeostasis, representing a promising strategy for both early intervention and treatment of established diabetes.

## Conclusion

In conclusion, sustained GLP-1 expression achieved through lentiviral delivery exerts distinct, stage-dependent effects on pancreatic regeneration, driving β-cell neogenesis and proliferation in the neonatal pancreas while partially reactivating regenerative pathways in adults. These findings highlight the potential of GLP-1–based gene therapy as a long-term strategy for restoring β-cell homeostasis and improving metabolic control in diabetes.

## Data Availability

All data generated or analyzed during this study are included in this article. Further inquiries may be directed to the corresponding author.

## Abbreviations

CK20: Cytokeratin 20
CMV: Cytomegalovirus
ELISA: Enzyme-Linked Immunosorbent Assay
GLP-1: Glucagon-like peptide-1
GLP-1R: GLP-1 receptor
GSIS: Glucose-Stimulated Insulin Secretion
H&E: Hematoxylin and eosin
INS: Insulin
IPGTT: Intraperitoneal Glucose Tolerance Test
ITT: Insulin Tolerance Test
MafA: MAF BZIP transcription factor A
MOI: Multiple of Injection
MPCs: Multipotent progenitor cells
Ngn3: Neurogenin-3
Nkx6.1: NK6 homeobox 1
OD: Obese-diabetic
Pdx-1: Pancreatic and duodenal homeobox 1
qPCR: Quantitative PCR
SD: Sprague Dawley
STZ: Streptozotocin
T2DM: Type 2 Diabetes
WPRE: Woodchuck Hepatitis Virus Posttranscriptional Regulatory Element

## References

[1] Ma Z, Zhang X, Zhong W, Yi H, Chen X, Zhao Y, Ma Y, Song E, Xu T (2023) Deciphering early human pancreas development at the single-cell level. Nat Commun 14:5354. 10.1038/s41467-023-40893-8 37660175 10.1038/s41467-023-40893-8 PMC10475098

[2] Lewis B, Mao J (2023) Development of the pancreas and related structures. The Pancreas, pp. 1–8. 10.1002/9781119876007.ch1

[3] Bonner-Weir S, Aguayo-Mazzucato C, Weir GC (2016) Dynamic development of the pancreas from birth to adulthood. Ups J Med Sci 121:155–158. 10.3109/03009734.2016.1154906 26998806 10.3109/03009734.2016.1154906 PMC4900072

[4] Dor Y, Brown J, Martinez OI, Melton DA (2004) Adult pancreatic β-cells are formed by self-duplication rather than stem-cell differentiation. Nature 429:41–46. 10.1038/nature02520 15129273 10.1038/nature02520

[5] Xu X, D’Hoker J, Stangé G, Bonné S, De Leu N, Xiao X, Van de Casteele M, Mellitzer G, Ling Z, Pipeleers D et al (2008) Beta cells can be generated from endogenous progenitors in injured adult mouse pancreas. Cell 132:197–207. 10.1016/j.cell.2007.12.015 18243096 10.1016/j.cell.2007.12.015

[6] Sanlioglu AD, Altunbas HA, Balci MK, Griffith TS, Sanlioglu S (2013) Clinical utility of insulin and insulin analogs. Islets 5:67–78. 10.4161/isl.24590 23584214 10.4161/isl.24590 PMC4204021

[7] Drucker DJ (2018) Mechanisms of Action and Therapeutic Application of Glucagon-like Peptide-1. Cell Metab 27:740–756. 10.1016/j.cmet.2018.03.001 29617641 10.1016/j.cmet.2018.03.001

[8] Portha B, Tourrel-Cuzin C, Movassat J (2011) Activation of the GLP-1 Receptor Signalling Pathway: A Relevant Strategy to Repair a Deficient Beta-Cell Mass. J Diabetes Res 2011:376509. 10.1155/2011/376509 10.1155/2011/376509 PMC3118608 21716694

[9] Tourrel C, Bailbé D, Meile MJ, Kergoat M, Portha B (2001) Glucagon-like peptide-1 and exendin-4 stimulate beta-cell neogenesis in streptozotocin-treated newborn rats resulting in persistently improved glucose homeostasis at adult age. Diabetes 50:1562–1570. 10.2337/diabetes.50.7.1562 11423477 10.2337/diabetes.50.7.1562

[10] Kodama S, Toyonaga T, Kondo T, Matsumoto K, Tsuruzoe K, Kawashima J, Goto H, Kume K, Kume S, Sakakida M et al (2005) Enhanced expression of PDX-1 and Ngn3 by exendin-4 during beta cell regeneration in STZ-treated mice. Biochem Biophys Res Commun 327:1170–1178. 10.1016/j.bbrc.2004.12.120 15652518 10.1016/j.bbrc.2004.12.120

[11] Hui H, Wright C, Perfetti R (2001) Glucagon-like peptide 1 induces differentiation of islet duodenal homeobox-1-positive pancreatic ductal cells into insulin-secreting cells. Diabetes 50:785–796. 10.2337/diabetes.50.4.785 11289043 10.2337/diabetes.50.4.785

[12] Tasyurek HM, Altunbas HA, Balci MK, Sanlioglu S (2014) Incretins: their physiology and application in the treatment of diabetes mellitus. Diabetes Metab Res Rev 30:354–371. 10.1002/dmrr.2501 24989141 10.1002/dmrr.2501

[13] Tasyurek HM, Altunbas HA, Balci MK, Griffith TS, Sanlioglu S (2018) Therapeutic Potential of Lentivirus-Mediated Glucagon-Like Peptide-1 Gene Therapy for Diabetes. Hum Gene Ther 29:802–815. 10.1089/hum.2017.180 29409356 10.1089/hum.2017.180

[14] Olgun HB, Tasyurek HM, Sanlioglu AD, Sanlioglu S (2019) High-Titer Production of HIV-Based Lentiviral Vectors in Roller Bottles for Gene and Cell Therapy. Methods Mol Biol 1879:323–345. 10.1007/7651_2018_150 29797007 10.1007/7651_2018_150

[15] Marzook A, Tomas A, Jones B (2021) The Interplay of Glucagon-Like Peptide-1 Receptor Trafficking and Signalling in Pancreatic Beta Cells. Front Endocrinol (Lausanne) 12:678055. 10.3389/fendo.2021.678055 34040588 10.3389/fendo.2021.678055 PMC8143046

[16] Müller TD, Finan B, Bloom SR, D’Alessio D, Drucker DJ, Flatt PR, Fritsche A, Gribble F, Grill HJ, Habener JF et al (2019) Glucagon-like peptide 1 (GLP-1). Mol Metab 30:72–130. 10.1016/j.molmet.2019.09.010 31767182 10.1016/j.molmet.2019.09.010 PMC6812410

[17] Wang KL, Tao M, Wei TJ, Wei R (2021) Pancreatic β cell regeneration induced by clinical and preclinical agents. World J Stem Cells 13:64–77. 10.4252/wjsc.v13.i1.64 33584980 10.4252/wjsc.v13.i1.64 PMC7859987

[18] Hu C, Chen Y, Yin X, Xu R, Yin C, Wang C, Zhao Y (2025) Pancreatic endocrine and exocrine signaling and crosstalk in physiological and pathological status. Signal Transduct Target Therapy 10:39. 10.1038/s41392-024-02098-3 10.1038/s41392-024-02098-3 PMC11825823 39948335

[19] Tasyurek MH, Altunbas HA, Canatan H, Griffith TS, Sanlioglu S (2014) GLP-1-mediated gene therapy approaches for diabetes treatment. Expert Rev Mol Med 16:e7. 10.1017/erm.2014.7 24666581 10.1017/erm.2014.7

[20] Movahednasab M, Dianat-Moghadam H, Khodadad S, Nedaeinia R, Safabakhsh S, Ferns G, Salehi R (2025) GLP-1-based therapies for type 2 diabetes: from single, dual and triple agonists to endogenous GLP-1 production and L-cell differentiation. Diabetol Metab Syndr 17:60. 10.1186/s13098-025-01623-w 39962520 10.1186/s13098-025-01623-w PMC11834518

[21] Eleazu CO, Eleazu KC, Chukwuma S, Essien UN (2013) Review of the mechanism of cell death resulting from streptozotocin challenge in experimental animals, its practical use and potential risk to humans. J Diabetes Metab Disord 12:60. 10.1186/2251-6581-12-60 24364898 10.1186/2251-6581-12-60 PMC7962474

[22] Wang C, Chen X, Ding X, He Y, Gu C, Zhou L (2015) Exendin-4 Promotes Beta Cell Proliferation via PI3k/Akt Signalling Pathway. Cell Physiol Biochem 35:2223–2232. 10.1159/000374027 25895469 10.1159/000374027

[23] Buteau J, Foisy S, Joly E, Prentki M (2003) Glucagon-like peptide 1 induces pancreatic beta-cell proliferation via transactivation of the epidermal growth factor receptor. Diabetes 52:124–132. 10.2337/diabetes.52.1.124 12502502 10.2337/diabetes.52.1.124

[24] Goode RA, Hum JM, Kalwat MA (2022) Therapeutic strategies targeting pancreatic islet β-cell proliferation, regeneration, and replacement. Endocrinology 164. 10.1210/endocr/bqac193 10.1210/endocr/bqac193 PMC9923807 36412119

[25] Xu G, Stoffers DA, Habener JF, Bonner-Weir S (1999) Exendin-4 stimulates both beta-cell replication and neogenesis, resulting in increased beta-cell mass and improved glucose tolerance in diabetic rats. Diabetes 48:2270–2276. 10.2337/diabetes.48.12.2270 10580413 10.2337/diabetes.48.12.2270

[26] Deng H, Yang F, Ma X, Wang Y, Chen Q, Yuan L (2020) Long-Term Liraglutide Administration Induces Pancreas Neogenesis in Adult T2DM Mice. Cell Transpl 29:963689720927392. 10.1177/0963689720927392 10.1177/0963689720927392 PMC7563804 32584149

[27] Sakhneny L, Khalifa-Malka L, Landsman L (2019) Pancreas organogenesis: Approaches to elucidate the role of epithelial-mesenchymal interactions. Semin Cell Dev Biol 92:89–96. 10.1016/j.semcdb.2018.08.012 30172049 10.1016/j.semcdb.2018.08.012

[28] Zhang Z, Hu Y, Xu N, Zhou W, Yang L, Chen R, Yang R, Sun J, Chen H (2019) A new way for beta cell neogenesis: transdifferentiation from alpha cells induced by glucagon-like peptide 1. J Diabetes Res 2019(2583047). 10.1155/2019/2583047 10.1155/2019/2583047 PMC6436340 31001561

[29] Zheng Z, Zong Y, Ma Y, Tian Y, Pang Y, Zhang C, Gao J (2024) Glucagon-like peptide-1 receptor: mechanisms and advances in therapy. Signal Transduct Target Ther 9:234. 10.1038/s41392-024-01931-z 39289339 10.1038/s41392-024-01931-z PMC11408715

[30] Friedrichsen BN, Neubauer N, Lee YC, Gram VK, Blume N, Petersen JS, Nielsen JH, Møldrup A (2006) Stimulation of pancreatic beta-cell replication by incretins involves transcriptional induction of cyclin D1 via multiple signalling pathways. J Endocrinol 188:481–492. 10.1677/joe.1.06160 16522728 10.1677/joe.1.06160

[31] Kumari P, Nakata M, Zhang BY, Otgon-Uul Z, Yada T (2018) GLP-1 receptor agonist liraglutide exerts central action to induce β-cell proliferation through medulla to vagal pathway in mice. Biochem Biophys Res Commun 499:618–625. 10.1016/j.bbrc.2018.03.199 29601817 10.1016/j.bbrc.2018.03.199

[32] Liu Z, Habener JF (2008) Glucagon-like peptide-1 activation of TCF7L2-dependent Wnt signaling enhances pancreatic beta cell proliferation. J Biol Chem 283:8723–8735. 10.1074/jbc.M706105200 18216022 10.1074/jbc.M706105200 PMC2417166

[33] Li Y, Hansotia T, Yusta B, Ris F, Halban PA, Drucker DJ (2003) Glucagon-like peptide-1 receptor signaling modulates beta cell apoptosis. J Biol Chem 278:471–478. 10.1074/jbc.M209423200 12409292 10.1074/jbc.M209423200

[34] Stoffers DA, Kieffer TJ, Hussain MA, Drucker DJ, Bonner-Weir S, Habener JF, Egan JM (2000) Insulinotropic glucagon-like peptide 1 agonists stimulate expression of homeodomain protein IDX-1 and increase islet size in mouse pancreas. Diabetes 49:741–748. 10.2337/diabetes.49.5.741 10905482 10.2337/diabetes.49.5.741

[35] Bulotta A, Hui H, Anastasi E, Bertolotto C, Boros LG, Di Mario U, Perfetti R (2002) Cultured pancreatic ductal cells undergo cell cycle re-distribution and beta-cell-like differentiation in response to glucagon-like peptide-1. J Mol Endocrinol 29:347–360. 10.1677/jme.0.0290347 12459036 10.1677/jme.0.0290347

[36] Drucker DJ (2013) Incretin action in the pancreas: potential promise, possible perils, and pathological pitfalls. Diabetes 62:3316–3323. 10.2337/db13-0822 23818527 10.2337/db13-0822 PMC3781450

[37] Pan FC, Wright C (2011) Pancreas organogenesis: from bud to plexus to gland. Dev Dyn 240:530–565. 10.1002/dvdy.22584 21337462 10.1002/dvdy.22584

[38] Nakamura T, Ito T, Uchida M, Hijioka M, Igarashi H, Oono T, Kato M, Nakamura K, Suzuki K, Jensen RT et al (2014) PSCs and GLP-1R: occurrence in normal pancreas, acute/chronic pancreatitis and effect of their activation by a GLP-1R agonist. Lab Invest 94:63–78. 10.1038/labinvest.2013.133 24217090 10.1038/labinvest.2013.133 PMC3879597

[39] Zhou Q, Brown J, Kanarek A, Rajagopal J, Melton DA (2008) In vivo reprogramming of adult pancreatic exocrine cells to beta-cells. Nature 455:627–632. 10.1038/nature07314 18754011 10.1038/nature07314 PMC9011918

[40] Zaret KS, Grompe M (2008) Generation and regeneration of cells of the liver and pancreas. Science 322:1490–1494. 10.1126/science.1161431 19056973 10.1126/science.1161431 PMC2641009

